# Opportunities and Challenges Arising from Holiday Clubs Tackling Children’s Hunger in the UK: Pilot Club Leader Perspectives

**DOI:** 10.3390/nu11061237

**Published:** 2019-05-30

**Authors:** Clare E. Holley, Carolynne Mason, Emma Haycraft

**Affiliations:** School of Sport, Exercise and Health Sciences, Loughborough University, Epinal Way, LE11 3TU Loughborough, UK; C.L.J.Mason@lboro.ac.uk (C.M.); E.Haycraft@lboro.ac.uk (E.H.)

**Keywords:** child, food poverty, food insecurity, holiday hunger, intervention, evaluation

## Abstract

With the school holidays being recognised as a high-risk time for children to experience food insecurity, there is a growing prevalence of school holiday initiatives that include free food. However, information is lacking into what constitutes effective practice in their delivery, and how this can be evaluated. This paper provides insight from individuals who implemented a pilot of a national project which provided free food for children at UK community summer holiday sports clubs in 2016. Focus groups were conducted with all 15 leaders of the holiday clubs that participated in the pilot to understand: *(1) what opportunities are provided by community holiday sports clubs which include free food; (2) what challenges arose as a result of offering free food within a broader community holiday club sports offer*. Results indicate that offering free food at such clubs creates multiple opportunities for attending children, including: experiencing social interactions around food; enhancing food experiences and food confidence; and promoting positive behaviour. However, free food provision is associated with challenges including resource constraints and tensions around project aims. Future work should determine whether holiday clubs can positively impact children’s wellbeing and healthy eating.

## 1. Introduction

Food insecurity has been defined as “limited or uncertain availability of nutritionally adequate and safe foods or limited or uncertain ability to acquire acceptable foods in socially acceptable ways (e.g., without resorting to emergency food supplies, scavenging, stealing or other coping strategies)” [1]. While food insecurity in UK children has not been measured to date, a recent UNICEF report indicates that food insecurity is prevalent in the UK, with 20% of children aged under 15 years old living with a respondent who is moderately or severely food insecure and 10% living with a respondent who is severely food insecure [2]. Research asserts that children who experience food insecurity are less likely to eat fruits, vegetables and brown bread, and are more likely to consume unhealthy foods such as chips and hamburgers [3]. Children who experience food insecurity are also likely to experience holiday hunger; the phenomenon by which children (particularly those who rely on free school meals during term-time) fall into a nutritional and calorie deficit in the school holidays [4,5].

Holiday hunger is likely to have a detrimental effect on school children’s educational performance, health and wellbeing. Although there are common issues with measurement of changes in educational attainment, gaps in attainment between US children in high and low poverty schools which increase across the summer have been reported [6], with summer learning loss also being evidenced among low socioeconomic status (SES) school children in the UK [7]. Moreover, food insecurity is associated with poorer health status and emotional wellbeing [8]. For example, 6-year-old to 11-year-old children from food insecure families demonstrate difficulty getting on with other children and are more likely to have seen a psychologist [9]. 

While free food services are increasingly in demand in the UK [10], there can be an associated social stigma that can inhibit families from utilising them [11]. However, free food provision integrated within holiday clubs presents an opportunity to encourage more individuals to attend these clubs while removing the social stigma associated with attending other free food services [12]. Creating holiday food provision within local communities therefore not only facilitates social support, which has a well-established relationship with health and wellbeing [13], but also ensures that the provision is accessible. This will likely maximise both the utility and the uptake of such provision. Tackling holiday hunger can lessen the nutritional deficit experienced by a significant proportion of UK children [14]. The disparity in educational attainment, health and wellbeing associated with food insecurity may also be reduced, while utilising this opportunity to engage the population in physical activity and other community-based activities. 

Despite there being an increasing number of holiday clubs across the UK attempting to tackle holiday hunger, there is incredibly limited research into the operational aspects and effectiveness of these clubs [5], and there is no published literature on similar projects in other countries of the developed world [15]. Previous qualitative research suggests that holiday breakfast clubs in the north-west of England and Northern Ireland may have positive social, nutritional, educational and financial impacts for those attending [12,16]. In further qualitative research, school holiday club staff asserted that holiday food provision is needed and that clubs can provide access to food and other activities including social and informal learning opportunities [17]. However, free food provision at community holiday sports clubs has not previously been examined and an in-depth analysis of the challenges that food provision presents is yet to be conducted.

Holiday clubs typically develop organically and are context specific and therefore operate without a fixed format, which presents challenges for robust evaluation work. Moreover, food insecurity is a multi-faceted issue, with multiple consequences, and there is variance in which aspects are targeted by holiday clubs. A recent systematic review has suggested that the evidence base regarding food insecurity interventions is mixed, has methodological limitations and that evaluations may be missing key areas of impact [15]. With this in mind, more information is needed on how the effectiveness of holiday projects which include free food can be evaluated, and how any potential impact can be assessed in future evaluation work. 

The current study sought to distil learning from the experiences of the 15 leaders who organised and delivered the holiday clubs which participated in the StreetGames Fit and Fed programme pilot (community holiday sports clubs including free food provision). The study sought to address two research questions: *(1) what opportunities are provided by holiday sports clubs which offer free food in disadvantaged communities; (2) what challenges arose as a result of offering free food within holiday sports clubs in disadvantaged communities*. Exploration of these questions was undertaken with the goal of informing further research to support the future success of the Fit and Fed project, and other holiday clubs that incorporate sport and food.

## 2. Methods

### 2.1. Ethics

The study was conducted in accordance with the Declaration of Helsinki, and Ethical clearance was obtained for this study from the Loughborough University Institutional Review Board.

### 2.2. Participants

Participants were the 15 leaders who organised and ran the pilot holiday clubs that were delivered through the Fit and Fed project over the school summer holiday period in 2016. These clubs took place across multiple counties within the UK, with the majority of these clubs (77%) taking place in the top 20% of deprived neighbourhoods in England and Wales as categorised by the English and Welsh indices of deprivation [18,19]. 

### 2.3. The Fit and Fed Programme

Fit and Fed is a national project run by the charity StreetGames. StreetGames utilises doorstep sport to achieve positive change in the lives of children from disadvantaged communities [20]. In response to large numbers of children who attend their neighbourhood youth sport opportunities experiencing holiday hunger, StreetGames developed the Fit and Fed project to provide free meals to children within disadvantaged community holiday sports clubs across the UK. The programme aims to tackle three main inequalities—holiday hunger, isolation, and inactivity—by providing food alongside the opportunity to participate in sporting activities and physical activity. Clubs do not operate under a fixed format, but rather implement their activities in the format appropriate for their club and the available resources. In its initial (pilot) year, the Fit and Fed project comprised 33 holiday clubs overseen by 15 holiday club leaders. The research team are entirely independent from StreetGames and the Fit and Fed project. 

### 2.4. Recruitment

After the school summer holiday in 2016, the 15 pilot club leaders were invited to participate in a feedback day to share their experiences of the pilot and ideas for the future progression of the programme. All leaders of pilot clubs attended the feedback day. One element of this feedback day was focus groups to capture club leaders’ insights based on their diverse pilot experiences.

### 2.5. Focus Groups

Written informed consent was obtained from participants before the onset of the focus group, with individuals informed of their right to withdraw at any point. Focus groups were conducted to create dynamic discussions between participants. In order to ensure sustained discussion at the same time as allowing space for all members to participate, the participants were split into two focus groups [21]. Group one had seven participants and lasted for 33 min and group two had eight participants and lasted for 44 min. 

The focus groups were digitally recorded. They were facilitated by the lead author (CH) who posed a series of questions compiled by the research team. The questions were derived from discussions within the research team as well as a review of the literature. 

### 2.6. Descriptive Information about the Holiday Clubs

After participating in the focus groups, pilot club leaders completed a questionnaire to provide information on the number, gender and age of children attending their clubs, the frequency and duration of the club sessions and the food provided. 

### 2.7. Analysis

Focus group recordings were transcribed verbatim and a thematic analysis was undertaken, following Braun and Clarke’s guidance [22]. As this is an exploratory study, coding was undertaken both with an inductive and deductive approach, where data were coded with research questions in mind in order to generate the resultant themes. The research questions were: *(1) what opportunities are provided by holiday sports clubs which offer free food in disadvantaged communities; (2) what challenges arose as a result of offering free food within holiday sports clubs in disadvantaged communities?* First, the lead author fully immersed themselves in the data. Second, sections of the transcripts recognised as meaningful in relation to the study aims were identified and manually labelled with an appropriate code. Once both transcripts had been coded, the third step of grouping codes according to a common theme was undertaken. This resulted in a set of related themes with distinct conceptual meaning. 

Research team members were involved in a discussion of the coded items to facilitate the creation of themes. Two of these members (C.M., E.H.) had not read the full transcripts of the focus groups and as such were judged to be suitable unbiased consultants. The reliability of the analysis was confirmed by a second, independent researcher (BAJ) who performed an analysis on 20% of the transcripts. The second coder used their own coding scheme, corroborated the themes that were identified by the primary researcher, and did not identify any further themes. This method of assessing reliability of the analysis is recognised as appropriate for a thematic analysis and has been widely used in previous research [23]. 

## 3. Results

### 3.1. Descriptive Information about the Holiday Clubs

Descriptive information about the frequency and duration of the holiday clubs is presented in Table 1. There was variability in the number of weeks that the clubs ran for, the number of sessions offered per week, and the duration of these sessions. Most clubs ran for five or more weeks, with some running once a week and some running every day. The profile of the child attendees also varied between clubs. However, 85% of attendees across clubs were aged 13 years or under, and slightly more attendees were male than female. 

Group leaders reported on the foods offered during the holiday club sessions across the summer (Table 2). All clubs provided sandwiches, while almost half provided a hot meal. Most clubs provided some form of fruit, while almost half provided a form of salad or vegetable. In addition, 53% of clubs offered a cooking or food related activity for the children to engage in, alongside food provision.

### 3.2. Thematic Analysis

Pilot club leaders’ insights from the project were explored in relation to two research questions: *(1) what opportunities are provided by holiday sports clubs which offer free food in disadvantaged communities; (2) what challenges arose as a result of offering free food within holiday sports clubs in disadvantaged communities?* For each of the two research questions, several themes were identified. These are discussed in detail in the following sections.

### 3.3. Research Question One: What Opportunities are Provided by Holiday Sports Clubs That Offer Free Food in Disadvantaged Communities?

The comments of the pilot club leaders revealed a number of different opportunities arising from the inclusion of the food offer at community holiday sports clubs. These opportunities were: promoting engagement, alleviating food scarcity, enhancing children’s food experiences, increasing ‘food confidence’, promoting social experiences with food and promoting positive behaviours. 

#### 3.3.1. Opportunity 1: Promoting Engagement

Almost all pilot club leaders identified that providing free food promoted children’s engagement with the holiday clubs. Leaders perceived that free food was a motivator for attendance at the sports clubs: *”the last session where we provided food was the busiest one”, “I think some people attended ours just for the free food”*; and that the provision of food increased the number of children attending their clubs: *”I think the food was a massive attraction and the numbers [of attendees] were better than [for previous] stuff we’d put on”*. These comments indicate that providing food can be an important incentive to encourage participation in projects that may deliver a range of positive health outcomes for children. Furthermore, providing universal free food for all attendees at the holiday clubs promoted engagement and removed the potential for the stigma associated with projects which seek to solely alleviate food insecurity: *”We were just a sports academy so that was it, other than Fit and Fed it was just a sports academy, there was no stigmatisation on anything else”*.

#### 3.3.2. Opportunity 2: Alleviating Food Scarcity

Pilot club leaders perceived the inclusion of the food offer to be important for addressing food scarcity related specifically to the school holiday period (holiday hunger). Pilot club leaders reported that parents face additional financial pressure in the school holidays: *”it costs more to live during the school holidays, and they’re preparing to go back to school in September; maybe school uniform is too small, got to get new…”*. These discussions suggested that families were forced to make decisions about how to prioritise their spending to afford the necessities. Some pilot club leaders also commented that food scarcity decreased the amount of food that parents allowed themselves to eat to provide food for their children: *”I see mums that don’t eat, because they don’t want to take the food out of the kids’ mouths, because that’s the only food that they’re gonna get”*.

#### 3.3.3. Opportunity 3: Enhancing Children’s Food Experiences

In addition to restrictions on the quantity of food available, pilot club leaders were concerned about the quality of food that was available to families within the targeted communities as a result of lack of money and a lack of access to more nutritionally appropriate (expensive) food: *”there’s no transport, there’s no finance – it’s difficult”*. It was recognised by pilot club leaders that financial issues impacted parents food provision, with them reporting that children and families opted to buy foods with lower nutritional value which were often cheaper and more readily available than healthy foods: *”some of the chicken places charge £1! For chicken and chips! A parent is going to look at that and go, do you know what, I’ve got a £1 for that but I can’t go shopping and spend £5 to do a meal”*. 

Pilot club leaders perceived there to be opportunities for the holiday clubs to enhance children’s experiences with food, but in doing so they demonstrated some stereotyped views on children’s prior food experiences. Pilot club leaders made assumptions around what they perceived to be evidence of a restricted diet: *”some kids don’t even know what tomatoes and cucumbers are!”, ”The kids in Blackpool didn’t even know you could get red apples, because mum and dad just always buy green. ‘What’s that?!’ ‘It’s an apple.’ ‘Don’t be so stupid, it’s red!’”*. Moreover, pilot club leaders reported witnessing child attendees choosing foods which were perceived to be ‘unhealthy’ when given a free rein: *”They’d come back with a two-litre bottle of something blue or like loads of bags of crisps and loads of sweets”*.

#### 3.3.4. Opportunity 4: Increasing ‘Food Confidence’

Related to the opportunity to alleviate food scarcity is the opportunity to increase ‘food confidence’. Pilot club leaders indicated that some attendees were very reluctant to try new foods that were not familiar to them, and they attributed this to children having a lack of confidence to try new foods: *”You’re all talking about the nutritional thing, we didn’t even do that. It was food confidence for us, it was some of these kids thinking ‘what’s that?!’”*. Some club leaders adopted innovative strategies to try and address this lack of food confidence by developing games and activities to encourage children to try different foods, with fruit and vegetable consumption being particularly targeted. Strategies included use of a smoothie bike where participants’ pedal power enabled the ingredients to be converted into smoothies: *”they were making these green smoothies with like spinach, banana, and they were tasting them like ‘well actually, that’s really nice, have that!’”*. Pilot club leaders also described how getting children involved in food preparation encouraged them to try foods they would not otherwise: *“it worked so well [using a bike powered blender] because where the kids…wouldn’t have eaten a banana, chuck it in milkshake, and because they’d done the pedalling themselves, they enjoyed it”*. The way in which food was prepared and delivered was noted as impacting on participants’ willingness to try new foods. For example: *”some of the coaches were saying to me that fruit was better received if it was chopped up to small bite sized pieces than it was if you gave them a whole apple”*. One pilot club leader suggested that this approach mirrored supermarket moves to sell prepared fruit which they sell at a premium price. Other strategies included quizzes and ‘bush tucker trials’ (eating challenges that appear in the popular television programme ‘I’m a celebrity…get me out of here’). 

#### 3.3.5. Opportunity 5: Promoting Social Experiences with Food

Pilot club leaders discussed several ways in which they provided opportunities to promote children’s social experiences with food. Some of the projects, particularly those that adopted a multi-partner delivery model which was accompanied by greater resourcing, deliberately engaged children in the preparation of the food that was eaten. This approach was seen as having the advantage of being an effective way of engaging parents and other family members in both cooking and the physical activity which was offered at clubs: *“I think it brought everyone into a whole new element because who doesn’t like to prep food, cook food and eat food? So, we called our project [name of project] … we had babies coming in, parents with the rest of the family coming in”*. 

A number of pilot club leaders mentioned the significance of sitting around the table when eating and this was something that was clearly considered to be both important and impactful by some club leaders: *”We always had a dessert as well because … it was a way of keeping them at the table and having that kind of meal environment”*. This is an interesting finding as this indicates again that pilot club leaders aspired to do more than just ensure children were not going hungry during the school holidays. Pilot club leaders described attempts to create a meal which they clearly associated as being a valuable and positive experience for participants and something that some leaders assumed may not happen at home for these children. 

#### 3.3.6. Opportunity 6: Promoting Positive Behaviours

Some pilot club leaders indicated that for some of the attendees at Fit and Fed, the provision of food impacted positively on children’s behaviour: *“there were changes in the behaviour of the young people because they were then fed, and obviously…they’re not then as lethargic, they’ve got their energy to talk and do things”*. Furthermore, pilot club leaders described improvements in both children’s mood as a result of being fed: *“a hungry child is an angry child”*; and also in their concentration: *”because they haven’t got to worry where their next meal is coming from they can concentrate more”*. Pilot club leaders also described changes in mood as altering the perceptions that other people may have of the young attendees: *“they’ll probably have been told oh you know ’moody young kid’, ‘moody teenager’, ‘always causing trouble again’, whereas there’s an actual reason for it, and those are the things that we can eradicate, and they can now be seen in a whole different light”*.

### 3.4. Research Question Two: What Challenges Arose as a Result of Offering Free Food Within Holiday Sports Clubs in Disadvantaged Communities?

The focus clubs uncovered five different overarching challenges arising from including free food at the holiday clubs. These challenges were: food provision, other resource constraints, the age of participants, staff attitudes and nutritional understanding and peer pressure.

#### 3.4.1. Challenge 1: Food Provision 

The discussions with pilot club leaders indicated considerable variation in the food that was provided at the different Fit and Fed projects. The availability of food was determined by a range of different factors but was perhaps most heavily influenced by the financial resources available: *”we didn’t have the funding to be able to go and get a lot of these things [foods], I mean we kept it basic”*. Issues with obtaining food supplies from big companies were also evident: *”we went to [name of supermarket] and they wanted nothing to do with it”*; and similarly, from food banks, who had insufficient supplies: *”we weren’t getting there early enough so all the other projects were getting the good stuff”*. Lastly, the available facilities dictated the kinds of food that clubs could provide and led to compromises: *”We didn’t have a cooker though, so we just sort of made sandwiches”*.

#### 3.4.2. Challenge 2: Other Resource Constraints

Pilot club leaders also described challenges related to other resources including a lack of staff: *”if we’d got 40 kids turning up and we’ve only got two [sport] coaches then we’ve got a safeguarding issue on our hands”*. Limited capacity and ensuring the health and safety of children and children at the facilities was also identified as a challenge: *”if it was bad weather…we could literally have no more than 30 odd kids in there, so we were a bit restricted”*. 

#### 3.4.3. Challenge 3: Age of Participants 

The majority of the club leaders in the pilot stated that they had predominantly engaged younger children (5–11) despite some staff suggesting that older children may have a greater need for a food offer: *“we work with what we class as the forgotten group, the 11–14 year olds, because if you’re younger than that someone will help you out, and if you’re older than 14 where we’re from you’re already making money”*. 

#### 3.4.4. Challenge 4: Staff Attitudes and Nutritional Understanding 

It was clear from the focus groups that staff had varying attitudes towards what constituted healthy eating, what children should be eating, and how they should be encouraged to eat healthily. Fruit and vegetables were generally perceived as foods to be encouraged by all staff: *”We tried to build it into every meal so…we had a main meal and a dessert so [in] one or the other there’d be either fruit or veg in both. Usually in every meal”*. Some staff were much more positive about participants accessing food high in sugar (e.g., desserts, sweets) than other staff: *“We always had a dessert as well.”, “One of our most popular meals was the ice cream, little cones, because they came up for seconds”, “We do give the kids treats like that as well as the fruit”*. 

Some pilot club leaders were traditional in their attempts to encourage children to eat healthy food as they discouraged participants from actions which limited the food that was eaten. These staff expected participants to eat the food they were provided with and were reluctant to adapt the food on offer to tailor for individual preferences: *”They were requesting ’can I have that without salad?’, [and we were saying to them] ’No, you can’t!’”*. Furthermore, some pilot club leaders were adamant that the food on offer needed to be ‘healthy’, while for other club leaders the need to tackle ‘food shortage’ was of primary importance: *“The most important thing is that they’re full, and the second most important thing is well if we can make things better then that’s great”*. This tension was not easily reconciled: *“We knew that parents and children wanted those normal, everyday nice foods, and part of us, our heartstrings were saying you know if these kids haven’t had a meal then they should have something they can enjoy as well that is not too radical for them, but on the other hand we should be promoting healthy eating”*.

#### 3.4.5. Challenge 5: Peer Pressure

Pilot club leaders highlighted that children’s eating behaviour was influenced by peers. This peer influence could be positive: *”they would kind of serve each other smoothies and they really liked that”, but it could also be a challenge: “we’d got them sandwiches, and they sat and picked all of the salad out of it, put it in the bin, closed the sandwich again…I am talking every single one of them would sit there and take the [salad] out”*.

## 4. Discussion

This study aimed to distil learning from the experiences of 15 pilot club leaders who provided free food for children attending community holiday sports clubs across the UK. It utilised the insights of leaders of the clubs who participated in the pilot project to determine: *(1) what opportunities are provided by holiday sports clubs which offer free food in disadvantaged communities; (2) what challenges arose as a result of offering free food within holiday sports clubs in disadvantaged communities*.

Pilot club leaders reported that providing free food as part of their holiday clubs promoted attendees’ engagement with the clubs. Children’s participation in sport can have multiple positive outcomes [24], with recreational sport developing children’s self-confidence, communication and teamwork skills, and willingness to challenge themselves [25]. Moreover, club leaders identified that embedding food provision within these sport contexts reduced the stigma of attending a free food initiative, supporting assertions made in previous research and highlighting the strength of this approach [12]. With UK government funding for holiday clubs being contingent on providing daily physical activity alongside free food [26], such opportunities for positive outcomes and reduced stigma are increasingly prevalent. 

Pilot club leaders reported opportunities for increasing food confidence, as well as enhancing children’s food experiences. This belief was rooted in a perception that participants had access to both a limited quantity and a limited range of food during the school holidays. Staff also reported that these issues were compounded by participants lacking food confidence and therefore self-limiting the food they chose to eat, as well as by the social influences on children’s food choices - notably the influence of peers. As diets low in fruit and vegetables are associated with an increased risk of numerous non-communicable diseases [27], holiday clubs that provide opportunities to diversify children’s diets and improve eating behaviours have the potential to prevent the development of disease and ill health. With this in mind, future research is needed to evidence whether holiday clubs do improve children’s food confidence and eating behaviours.

Although comments from club leaders identified opportunities to enhance children’s food experiences and food confidence, not all clubs capitalised on this opportunity. Food provision was a significant challenge for clubs, and while most (93%) clubs offered fruit, just 43% offered vegetables. While it is important that children experiencing food insecurity are given energy-dense foods which will help to attenuate their calorie deficit, the nutritional deficit they experience should also be tackled by such community interventions if potential health benefits are to be realised. This would require implementing a minimum standard of food which provides child attendees with the most appropriate foods in terms of both macro- and micro-nutrients, in order to achieve maximal health benefits. While this was beyond the reach of the clubs who participated in this pilot, this is a new requirement of the Department for Education’s recently pledged funding for holiday clubs across the UK, where all food provided at clubs (including snacks) must meet the school food standards [26]. Future work should seek to determine how the challenges of providing nutritionally balanced foods within holiday club contexts can be overcome.

A further opportunity described by pilot club leaders was promoting social experiences with food, with some clubs able to engage children in food preparation and/or enjoy shared mealtimes around a table. Previous research has suggested that involving children in cooking can increase children’s consumption of vegetables as well as meals, highlighting the potential of this opportunity [28,29,30], and the impact of this should be explored in future research. Moreover, shared mealtimes are associated with children having a reduced risk of substance abuse and paediatric obesity, promoting language development and higher academic achievement [31] and better psychological wellbeing [32], highlighting the value of these new experiences. With this in mind, wellbeing may be one appropriate indicator of intervention success in future research. 

Some pilot club leaders outlined opportunities for the provision of free food to promote positive behaviours. Food insecurity has been associated with increased odds of substance abuse and behavioural, anxiety, and mood disorders during the previous year [33]. Moreover, Maslow’s hierarchy of needs suggests that children are unable to perform higher order behaviours, such as respect for self and others, until lower order needs, such as hunger, are satisfied [34]. Here, hunger shows a prepotency over behaving in a socially acceptable way. In line with one leader’s statement that “a hungry child is an angry child”, it therefore seems plausible that short-term alleviation of food insecurity could be related to improvements in children’s moods. This suggests that by tackling holiday hunger, projects may have the potential to reduce the likelihood of antisocial behaviour among some attendees who have a propensity towards these behaviours. Further work is needed to explore this.

Pilot club leaders widely reported that resources were a challenge for community holiday clubs and that these restricted the food provision and the number of children each club could accommodate. This is also supported by the descriptive information gathered from the clubs. The number of weeks that the clubs ran for and the frequency of sessions they offered varied greatly in accordance with resource constraints as a lack of resources unfortunately appears to be common. However, with the UK government’s Department for Education (DfE) pledging £9 million in funding for holiday clubs for children in disadvantaged communities, there is optimism that these resource issues can be minimised for many clubs. 

This paper has focused primarily on understanding what happens when free food is offered alongside physical and social activities to create a broader provision for children. Such provision potentially offers better value for money in that multiple outcomes can be achieved simultaneously. However, this model creates challenges in terms of evaluating the effectiveness of the project because the aims are complex and inter-linked. 

It was clear from the experiences discussed in the focus groups that there was considerable diversity in terms of what the projects sought to achieve within their clubs and also considerable variation in how the clubs undertook the Fit and Fed pilot project. They varied in terms of inter alia partners involved, settings, delivery approach/activities, age range of participants, reach and aims of the club. Such variation raises questions about what constitutes effectiveness and how this can best be measured, including whether success of community projects should be measured according to local community aims, or the aims of broader public health programmes. Indeed, whilst many of the clubs included in this study met the DfE’s minimum standards required for 2019 publicly funded UK holiday clubs, it also underlines the complexities of achieving (and assessing) the UK government’s aspiration of locally coordinated free holiday activities and healthy food for disadvantaged children. 

While most projects aimed to ensure that children did not experience ‘holiday hunger’, they adopted different ways of addressing the issue of food scarcity. Indeed, most projects were concerned with much broader food aims related to the quality of participants’ food experiences. In addition to tackling food scarcity, most projects also attempted to promote ‘healthy’ eating, but again, there was considerable variation in terms of what was considered as ‘healthy’ eating and how this outcome could best be achieved. Moreover, some projects promoted intake of food regardless of nutritional composition, which may exacerbate unhealthy eating behaviours in some children. 

The novel contribution of this paper includes highlighting the challenges that exist regarding developing a robust evidence base for projects that are not uniformly implemented, where evaluation of impact needs to be aligned with the overarching aims of these projects. The aspirations for including a food offer within clubs were not clearly articulated by many clubs. Developing a more robust evidence base will require not only the articulation of these intended outcomes, but also a more advanced understanding of how these outcomes can best be delivered for which children. Having unpacked the insights of holiday club leaders on the opportunities provided by holiday clubs, this paper provides novel insight into previously unexplored areas of potential impact across a range of outcomes associated with child wellbeing and healthy eating, including dietary variety, dietary intake, food choice and willingness to try new foods. 

This study provides an important account of pilot club leaders’ perceptions of the opportunities that community projects which offer free meals during the school holidays can provide. By gathering qualitative data, a detailed picture of the potential impact of these projects was developed. While the qualitative methods employed here have clear strengths, it must be acknowledged that the evidence collected for this study is experiential and future research should seek to further explore the assertions made in these focus groups. For example, club leaders may have held stereotyped perceptions of the child attendees and, in turn, the opportunities created for these children by the holiday clubs. Future research is also needed which explores the views of children and families who engage in these projects, as those with the best knowledge of their own experiences [35]. The limited data gathered in this study prevents conclusions being drawn about the efficacy of a particular format of a successful club, or what a club must offer in order to be successful or what constitutes success for holiday hunger clubs. However, it does provide valuable insight into potential areas of impact to be explored in future research.

## 5. Conclusion

This study provides valuable insight into the opportunities presented by adding a free food provision to community holiday clubs, as well as the challenges this creates. With holiday hunger becoming a more prominent issue, projects that provide greater value for money by seeking to address a range of complex inter-related outcomes may be preferential to other models of intervention. However, progress in securing a strong evidence base about what works, why and for whom regarding holiday hunger is only in its infancy. Future research should seek to evaluate these projects according to these newly identified areas of potential impact around children’s wellbeing and healthy eating. Future research should also assess the appropriateness and benefits of recently introduced minimum standards for UK government projects of this nature, to ensure that projects are able to maximise the opportunities and benefits they provide for children and their families.

## Figures and Tables

**Table 1:** Descriptive information on the frequency and duration of the Fit and Fed pilot clubs (*n* = 15).

Holiday Club Information	Mean	Min	Max	SD
Number of weeks ran for	4.53	1.00	6.00	1.55
Number of sessions per week	2.63	1.00	5.00	1.23
Session duration (hours)	4.30	2.00	6.00	1.27
Percentage of male attendees	65.95	40.43	95.00	13.48

SD = standard deviation.

**Table 2:** Descriptive information on the number and percentage of Fit and Fed pilot clubs (*n* = 14 due to missing data) serving different types of food.

Food	*n* of Clubs Which Served Food	%
Sandwich/wrap	14	92.9
Hot meal	6	42.9
Crisps	5	35.7
Biscuits	7	46.7
Fruit	13	92.9
Salad/Vegetable	6	42.9
